# Recent Advances on the Role of ATGL in Cancer

**DOI:** 10.3389/fonc.2022.944025

**Published:** 2022-07-13

**Authors:** Renshuai Zhang, Jingsen Meng, Shanbo Yang, Wenjing Liu, Lingyu Shi, Jun Zeng, Jing Chang, Bing Liang, Ning Liu, Dongming Xing

**Affiliations:** ^1^ Cancer Institute, The Affiliated Hospital of Qingdao University, Qingdao, China; ^2^ Qingdao Cancer Institute, Qingdao, China; ^3^ School of Life Sciences, Tsinghua University, Beijing, China

**Keywords:** Adipose triglyceride lipase (ATGL), lipid metabolism, hypoxia, cancer, HIF-1

## Abstract

The hypoxic state of the tumor microenvironment leads to reprogramming lipid metabolism in tumor cells. Adipose triglyceride lipase, also known as patatin-like phospholipase= domain-containing protein 2 and Adipose triglyceride lipase (ATGL), as an essential lipid metabolism-regulating enzyme in cells, is regulated accordingly under hypoxia induction. However, studies revealed that ATGL exhibits both tumor-promoting and tumor-suppressing effects, which depend on the cancer cell type and the site of tumorigenesis. For example, elevated ATGL expression in breast cancer is accompanied by enhanced fatty acid oxidation (FAO), enhancing cancer cells’ metastatic ability. In prostate cancer, on the other hand, tumor activity tends to be negatively correlated with ATGL expression. This review outlined the regulation of ATGL-mediated lipid metabolism pathways in tumor cells, emphasizing the Hypoxia-inducible factors 1 (HIF-1)/Hypoxia-inducible lipid droplet-associated (HIG-2)/ATGL axis, peroxisome proliferator-activated receptor (PPAR)/G0/G1 switch gene 2 (G0S2)/ATGL axis, and fat-specific protein 27 (FSP-27)/Early growth response protein 1 (EGR-1)/ATGL axis. In the light of recent research on different cancer types, the role of ATGL on tumorigenesis, tumor proliferation, and tumor metastasis was systemically reviewed.

## Introduction

Lipid metabolism plays a vital role in cell death, growth, signaling, metabolism, and gene expression, and disruptions in lipid metabolism can likewise impact these processes (1, 2). As an important intracellular organelle for lipid storage, the formation and breakdown of lipid droplets (LDs) have important implications for lipid metabolism in cells. LDs are highly dynamic organelles in cells rich in lipids such as cholesterol and acylglycerol. The reprogramming of lipid metabolism in cancer cells results in the accumulation of LDs in cancer cells. The massive accumulation of LDs in non-adipocytes has now been recognized as a marker of cellular carcinogenesis. The abundance of LDs also promotes cancer cell proliferation, invasion, metastasis, and drug resistance (3). Like normal cells, tumor cells use LDs to cope with nutrient overload or nutrient deprivation in the tumor microenvironment (4). The synthesis and breakdown of LDs are precisely regulated in tumor cells (4). The lipid uptake and removal mechanisms, different lipid synthesis mechanisms, and lipid recycling mechanisms are used to meet the lipid requirements of tumor cells in different states (4). LDs can be used in cancer cells to ensure energy production and redox homeostasis, regulate autophagy, drive membrane synthesis and control its composition, and respond to lipid peroxidation and cellular iron death by regulating the storage and release of unsaturated fatty acids (4).

**Figure 1:**
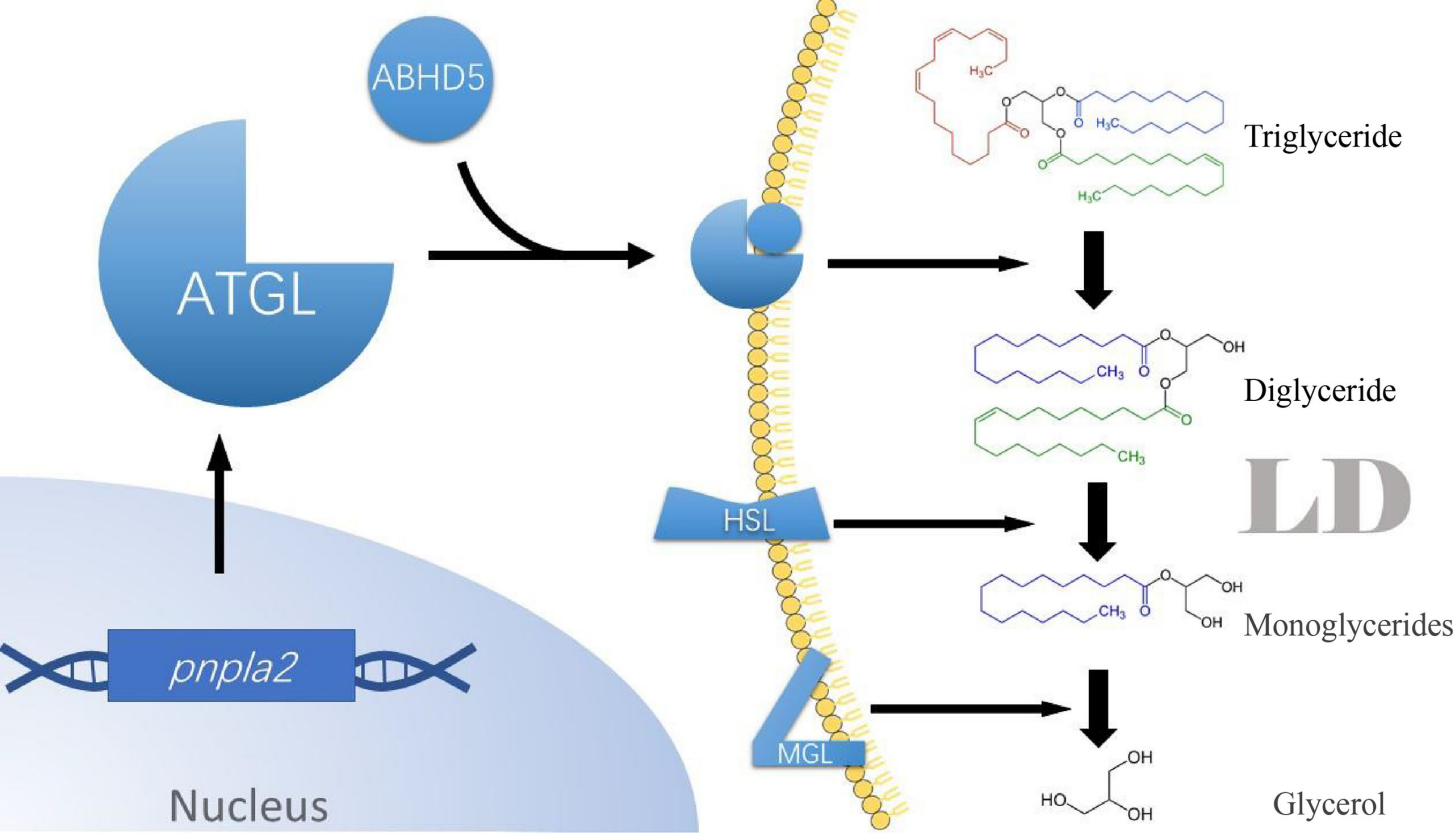
Series hydrolysis process of triglycerides by different lipases sitting in lipid droplets (LOs) membrane. ATGL binds to coactivator ABHDS and co-binds to the LD membrane. ATGL then hydrolyzes triglyceride to diglycerides and releases a molecu le of free fatty acids. HSL then hydrolyzes the diglycerides to glycerol monoesters and releases one molecule of free fatty acids. Finally, MGL hydrolyzes triglycerides and releases the last molecule of free fatty acids. ATGL, Adipose triglyceride lipase; ABHDS, 1-acylglycerol-3-phosphate 0-acyltransferase; HSL, Hormone-sensitive lipase; MGL, Monoacylglycerol lipase.

Related proteins involved in LDs formation include the perilipin protein family, ATGL, diglyceride acyltransferase (DGAT), and secretory phospholipase A (sPLA) (5–7). In cancer cells, all of these proteins are altered to varying degrees, especially ATGL. Intracellular phosphorylated lipases break down triglycerides (8) stored in LDs to release free fatty acids (FFAs) (9) involved in intracellular energy metabolism and signal transduction. These lipases include ATGL (10), hormone-sensitive lipase (HSL), and monoacylglycerol lipase (MGL). Among them, ATGL is the rate-limiting enzyme for triglyceride degradation. It is responsible for the breakdown of one molecule of triglycerides into one molecule of diglycerides and one molecule of FFAs. At the same time, HSL and MGL are accountable for the breakdown of diglycerides and monoglycerides, respectively (**Figure 1**). ATGL is encoded by the *Pnpla2* gene in the human body and has very high substrate specificity for triglycerides. The N-terminal of ATGL is a three (α/β/α) sandwich domain (residues 1-253). This segment contains a Patatin-like structural domain (residues 10-178), an α-helical structure (residues 10-24), and catalytic serine-aspartate duplexes (Ser 47 and Asp 166), which are essential in triglyceride substrate binding and triglyceride hydrolysis, respectively (11). ATGL contains hydrophobic lipid-binding stretches (residues 315-364) at its C-terminus, as well as two potential AMP-activated protein kinase (AMPK) phosphorylation sites (Ser 404 and Ser 428), which are responsible for the localization of ATGL on the LD (11). *In vivo*, perilipin A can regulate its activity through the coactivator 1-acylglycerol-3-phosphate O-acyltransferase (ABHD5) of ATGL (12). In addition, G0S2 can also bind competitively to ABHD5, thereby inhibiting ATGL activity. Defective ATGL in humans directly affects the cellular lipolysis process, leading to neutral lipid storage disease (13).

The availability of oxygen to cells within the tumor tissue decreases with increasing cell-vascular distance. In contrast, the disorganized vascular network within the tumor tissue and the distance between vessels are greater than the diffusion distance of oxygen (approximately 100-200 μm, depending on the oxygen content of the blood at the site versus the oxygen consumption rate of the surrounding tumor cells) leading to hypoxia in most areas within the tumor (14).To adapt to hypoxia, cancer cells change their metabolism from oxidative phosphorylation, the primary energy supply in normal cells, to glycolysis, known as the Warburg effect (15). In addition, several oncogenes, including *Ras*, *Src*, and *Myc*, also enhance the expression of glycolysis and glucose transport-related proteins, which promote the adaptation of tumor cells to the hypoxic environment (16, 17). The attenuation of oxidative phosphorylation also inhibits intracellular fatty acid β-oxidation to reduce oxygen consumption. Therefore, theoretically, the activity and expression of ATGL in cancer cells should be negatively correlated with the activity of cancer cells. Indeed, a decrease in ATGL protein levels can be observed in cancers such as human non-small cell lung cancer and pancreatic cancer, and *Pnpla2* is frequently reported to be absent in these cancer cells (18). Similarly, decreased levels of ATGL gene expression have been associated with lower survival rates in patients with cervical and gastric cancers. However, in some cancers, ATGL has a promotive effect on tumor progression (9, 19–21). Therefore, the role of ATGL in tumor cells is uncertain, and it might depend on the type of tumor and the environment in which the tumorigenesis is located. What is certain is that ATGL is a crucial player in regulating tumor metabolism under hypoxia (22). Recently, Rolando Vegliante et al. presented the role of the Peroxisome proliferator-activated receptor alpha (PPAR-α)/ATGL axis in cancer (23). However, ATGL regulates lipid metabolism in cancer cells in other pathways outlined in this review, namely, the HIF-1/HIG-2/ATGL axis, PPAR (PPAR-α and PPAR-γ)/G0S2/ATGL axis, and FSP-27/EGR-1/ATGL axis. Furthermore, we reviewed the recent research progress on the role of ATGL in different cancer types, hoping to shed light on future studies targeting ATGL for treating cancer.

## Regulation of ATGL in Cancer

### HIF-1/HIG-2/ATGL Axis

HIF-1 (HIF-1α and HIF-1β) was first reported by Semenza and colleagues in 1995 as an oxygen-sensitive transcription factor capable of responding to decreases in oxygen levels in the environment. Under normoxic conditions, the proline residues in HIF-1α are hydroxylated by the HIF-prolyl hydroxylase domain enzyme (PHD) and then degraded by ubiquitination of the von Hippel-Lindau protein (pVHL) (24–26). Under hypoxic conditions, PHD activity is inhibited, and HIF-1α is thus able to stabilize and translocate into the nucleus, where it binds to specific DNA hypoxia response elements (27) and activates the transcription of a large number of genes. The hypoxic environment to which tumor tissues are exposed leads to an upregulation of HIF-α expression in tumor cells (28). Furthermore, in addition to being induced by hypoxia, mutations in proto-oncogenes and tumor suppressors can also cause elevated HIF-1α expression (29). The upregulation of HIF-1α in clear stromal renal cell carcinoma results from VHL tumor suppressor, not hypoxia (30).

**Figure 2:**
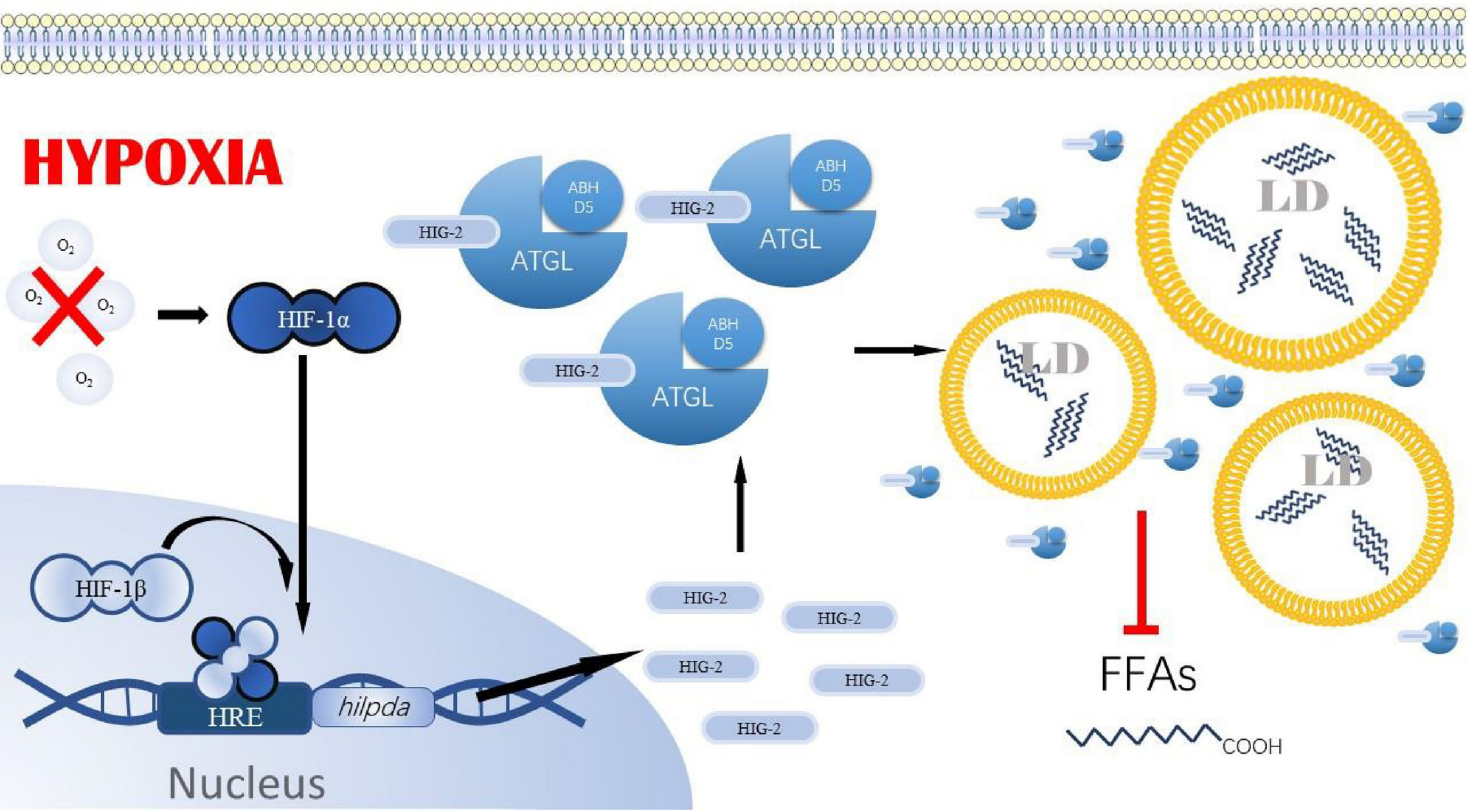
HIF-1/HIG-2/ATGL axis. Under hypoxia, HIF-1α- la can enter the nucleus of tumor cells and bind to HIF-1β, forming a stable dimer. This dimer binds to the HRE region upstream of hilpda, allowing tumor cells to express HIG-2 in large amounts. Even in t h e presence of ATGL binding to coactivator ABHDS, HIG-2 can still bind to ATGL, resulting in the inability of ATG L to anchor to the LD membrane and the inhibition of activity. Eventually, there is a significant accumulation of tri glycerides in tumor cells, resulting in lipid droplet accumulation. HIF-1α, Hypoxia-inducible factor I α; HIF-1β , Hypoxia-inducible factor 1 β . HRE, Hypoxia response element; ATGL, Adipose triglyceride lipase; HIG-2, Hvpoxia-inducible lipid droplet-associated. LD, Lipids droplet. FFAs, Free fanv acids.

HIG-2 a newly identified lipolytic regulator of cellular response to hypoxia in recent years, is encoded by *Hilpda*, a target gene of HIF-1. HIG-2 is expressed in various cells, including cancer cells, hepatocytes, adipocytes, and immune cells. Analysis of HIG-2 expression in the Cancer Genome Atlas database and the Pan-Cancer Data Center revealed that *Hilpda* is a marker of poor prognosis in tumor patients and positively correlates with increased infiltration of tumor-associated macrophages and immunosuppressive genes in the tumor microenvironment (31). Similarly, in pancreatic cancer cells, the bioinformatic analysis also revealed elevated expression of hypoxic lipid droplet-associated proteins (10). Moreover, in pancreatic ductal adenocarcinoma (PDAC) mouse model, HIG-2 was also found to increase the accumulation of LDs in cancer cells and promote the growth of cancer cells (32). Functionally, HIG-2 regulates fat storage in response to insufficient oxygen supply outside the cell or excess fatty acid content in the cell. The function of this protein is partially dependent on triglyceride hydrolase ATGL and triglyceride synthase DGAT. In a mouse hepatoma cell model, HIG-2 increased triglyceride synthesis and storage by stimulating DGAT1 (33). In another mouse model of colorectal cancer, knockdown of HIG-2 enhanced LDs degradation in cancer cells and promoted apoptosis in a Reactive oxygen species (ROS)-dependent manner, ultimately inhibiting the growth of tumor explants in mice. This enhanced effect of lipid degradation could be reversed by co-ablation of ATGL (34). It was confirmed by immunoprecipitation that HIG-2 could physically bind to ATGL (34). Further studies showed that both the interaction and co-localization of HIG-2 with ATGL on the LDs surface require a segment of the hydrophobic domain, LY(V/L) LG, which is also possessed by G0S2, a direct inhibitor of ATGL (34, 35). The above results suggest the presence of hypoxia-induced HIF-1/HIG-2/ATGL axis in tumor cells. Hence, we speculate that, possibly, tumor cells reduce Fatty acid β-oxidation through this axis to reduce oxygen consumption under hypoxic conditions. **Figure 2** illustrates the ATGL-mediated lipid metabolism pathway regulation by HIF-1 under hypoxia conditions.

### PPAR-γ/G0S2/ATGL Axis

G0S2 is the gene that controls the shift of the monocyte cycle from the G0 phase to the G1 phase (36, 37). The gene is highly conserved and has 78% identity in humans and mice. G0S2 was found to be a multifunctional protein involved in various intracellular biological pathways, including apoptosis, inflammation, metabolism, oxidative phosphorylation, and possibly even antitumor effect (38–42). G0S2 has been identified as a direct intracellular inhibitor of ATGL and is abundantly expressed in metabolically active tissues, such as adipose tissue, heart, skeletal muscle, etc. (43). G0S2 achieves triglyceride regulation by binding to ATGL to inhibit its hydrolysis of triglycerides. G0S2 inhibits the activity of ATGL even in the presence of the ATGL coactivator ABHD5, and the inhibitory effect of G0S2 on ATGL is dose-dependent and noncompetitive. Mechanistically, G0S2 possesses the same hydrophobic domain (HD) as HIG-2 and can bind to the patatin-like region of ATGL to inhibit its activity. In addition, tumor necrosis factor alpha (TNF-α) was found to stimulate ATGL-mediated lipolysis, which was also inhibited by G0S2 (44). In cancer cells, G0S2 can similarly regulate LDs lipolysis and promote LDs accumulation in cancer cells by inhibiting ATGL activity. Multiple bioinformatics analyses showed that G0S2 was highly expressed in pancreatic, breast, and rectal adenocarcinomas (10, 45, 46). In contrast, double knockout of *Pnpla2* and *Lipe* induced liposarcoma in mice, and abnormal expression of G0S2 was observed in double knockout liposarcoma tissues (47). Notably, knockdown of G0S2 produced tumor suppression, but this effect did not seem to be dependent on the ATGL inhibitory effect of G0S2 (42, 48).

On the one hand, G0S2 is involved in regulating cellular lipid metabolism by inhibiting its activity through direct binding to ATGL. On the other hand, G0S2 expression is also regulated by PPAR (PPAR-α, PPAR-β/δ, and PPAR-γ), a group of nuclear receptor proteins that play an essential role in regulating differentiation, development, metabolism, and tumorigenesis in higher organisms. PPAR-α and PPAR-γ have been extensively studied and found to be elevated in various cancer cells compared to normal tissues, such as breast cancer, colon cancer, liposarcoma, pancreatic cancer, and hepatocellular carcinoma (49). G0S2 is a target gene of PPAR that was first identified by Fokko ZANDBERGEN et al. (50). In 3T3-L1 fibroblasts, the G0S2 promoter was found to contain a functional PPAR response element, leading to its expression being regulated by PPAR. G0S2 was identified as a direct target gene of PPAR-γ by transactivation, gel shift, and chromatin immunoprecipitation assays. The stimulation of lipolysis in adipocytes by TNF-α was also caused by decreased G0S2 expression due to PPAR-γ inhibition (51). In addition, when Hep-G2 cells were treated with palmitate, PPAR-γ and G0S2 expression simultaneously elevated similarly (52).

**Figure 3:**
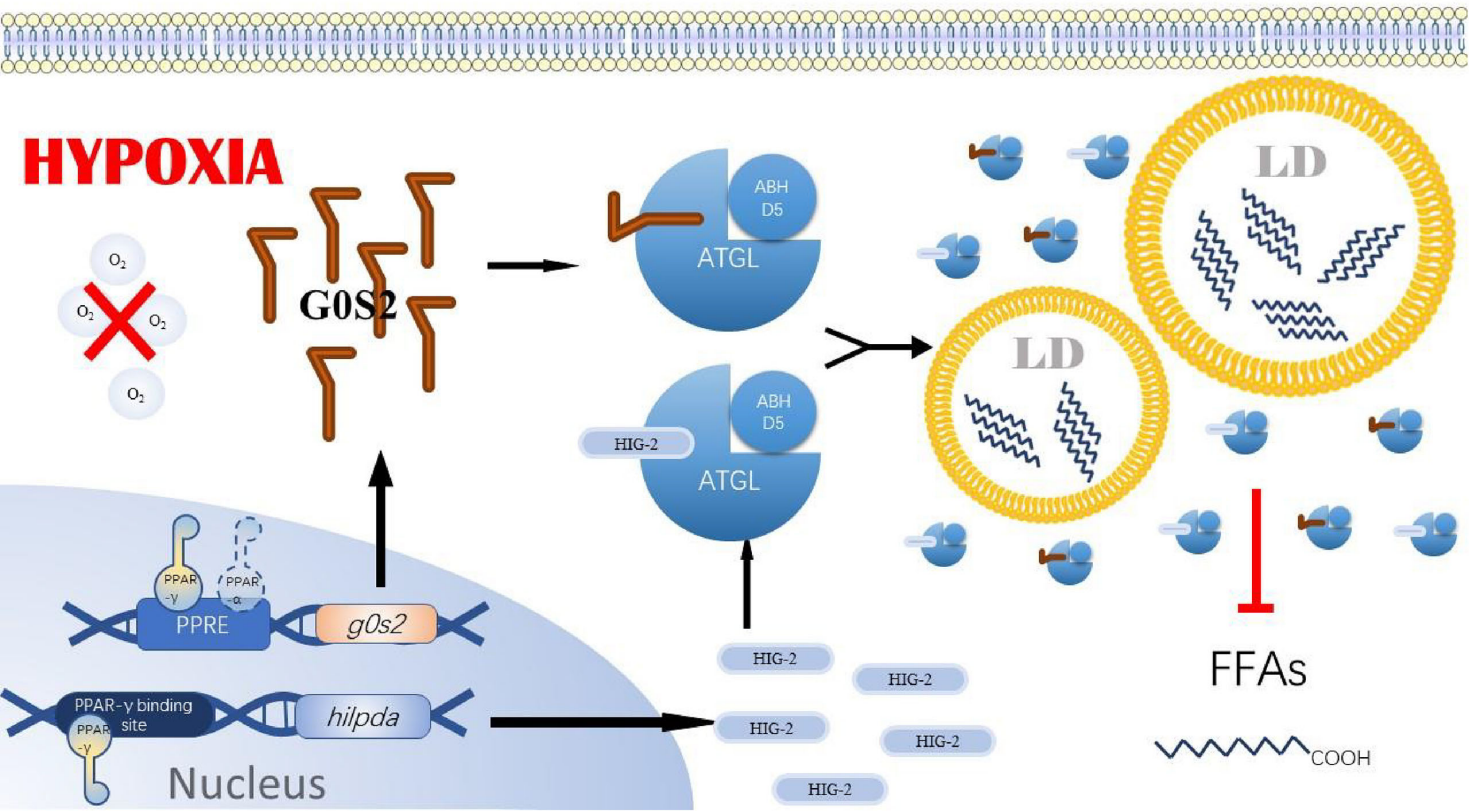
PPAR-y/G0$2/ATGL axis. In tumor cells under hypoxia, PPAR-gγ in cells binds to the PPRE region upstream of g0s2 and hilpda, promoting the expression of GOS2 and HI G-2. PPA R-α also binds to the PPRE region upstream of gOS2, promoting the expression of GOS2. gOS2 contains a similar segment of HD as HIG-2 and is also able to bind ATGL, inhibiting ATGL anchoring to the LD membrane and its activity. Ultimately, the overexpression of GOS2 and HIG-2 in tumor cells prevents ATGL from performing its typical hydrolytic role, leading to the accumulation of lipid droplets. PPAR-α: peroxisome proliferator-activated receptor a, PPA R-γ: peroxisome proliferator-activated receptoryγ, GOS2, GO/G1 switch gene 2; PPRE, PPAR response element; ATGL, Adipose triglyceride lipase; ABHDS, I­ acylglycerol–3–phosphate O acyltransferase; LD, Lipids droplet; FFAs, Free fatty acids14.

A critical PPAR-γ super-enhancer was found in human and mouse adipocytes near the HIG-2 gene, containing multiple conserved PPAR-γ binding sites (53). In contrast, in hepatocytes, the regulation of HIG-2 by PPAR-α was also demonstrated by transactivation and chromatin immunoprecipitation assays. Although these results were not confirmed in cancer cell lines, we believe that PPAR-α and PPAR-γ in cancer cells play a crucial role in hypoxia-induced reprogramming of lipid metabolism. Based on the studies of PPAR-α, PPAR-γ, G0S2, and ATGL in tumors, we speculate that under the induction of hypoxia in the tumor microenvironment, cancer cells increase the expression of PPAR-α and PPAR-γ, which in turn promote the expression of their target genes G0S2, to avoid the occurrence of required FAO pathway. As a direct inhibitor of ATGL, the high expression of G0S2 makes ATGL functionally inactivated in cancer cells, ultimately leading to the accumulation of triglycerides and LDs in cancer cells (**Figure 3**). We also found that although PPAR-γ expression is upregulated in many cancer cell lines, PPAR-γ coactivator Peroxisome proliferator-activated receptor gamma coactivator 1-alpha (PGC-1α) and PPAR-γ agonists have tumor-suppressive effects, both of which were able to upregulate FAO as well as mitochondrial oxidative phosphorylation processes. In addition, it is noteworthy that G0S2 can also promote F0F1-ATP synthase activity and increase cellular adenosine triphosphate (54) content (41). This may compensate for decreased fatty acid oxidation and decreased ATP content by increased glycolysis rate under hypoxia. In tumor cells, especially in cancer cell lines like breast cancer cells and lipoma cells where G0S2 expression is significantly upregulated compared to normal cells, it is not elucidated whether G0S2 also has a promoting effect on F0F1-ATP synthase. However, we believe that this effect is also present in cancer cells under a hypoxic microenvironment and positively impacts the survival of cancer cells.

### FSP-27/EGR-1/ATGL Axis

Both the HIF-1/HIG-2/ATGL axis and the PPAR-γ/G0S2/ATGL axis ultimately affect the release of fatty acids (FAs) from LDs by inhibiting the activity of ATGL proteins, and no studies have demonstrated that *Pnpla2*, the gene responsible for encoding ATGL, is in this process is regulated in this process. There are also no studies that clearly describe the regulation of *Pnpla2* in tumor cells. However, we found that in adipocytes, FSP-27 (also known as CIDEC) can cooperate with EGR-1, also known as ZNF268 (zinc finger protein 268) or NGFI-A (nerve growth factor-induced protein A) to suppress the expression of ATGL (55). In addition, FSP-27 is also able to reduce the release of FAs in LDs by directly inhibiting ATGL protein activity as G0S2 does with HIG-2 (56).

**Figure 4:**
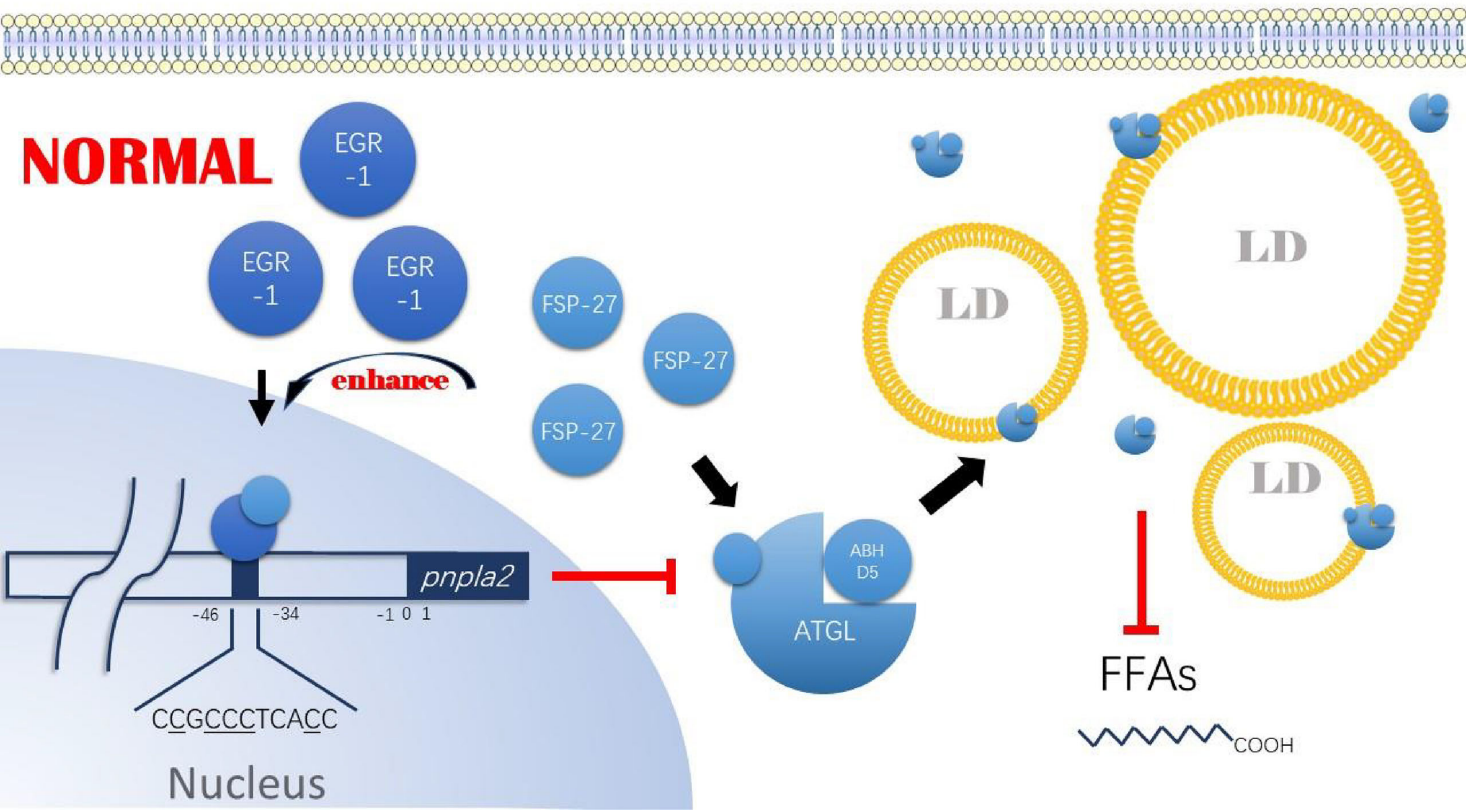
FSP-27/EG R-1 /ATG L axis. Under nom1al conditions, FSP-27 can bind to the core structural doma in of ATGL, aa 120-220, and inhibit its triglyceride hydrolysis function. Tn addition, FSP-27 can promote the bin di ng ofEGR-1 to the -45/-34 region upstream of pnpla2, thereby inhibiting ATGL expression. Ultimately, the intracellular ATGL content decreases, and its activity is reduced, allowing intracellular lipid droplet accumulation. FSP-27, Fat-specific protein 27; EG R-1, Early growth response protein I; ATGL, Adipose triglyceride lipase; A-BH DS, 1–acylglycerol-3- phosphate 0-acyltransferase; LDs Lipids droplet; F'FAs, Free fatty acids.

In cells, FSP-27 is mainly involved in LDs morphology regulation (57–59). Depletion of FSP-27 in adipocytes leads to fragmentation of LDs (60, 61). In contrast, when FSP-27 is overexpressed, it promotes the fusion of LDs (62) and the exchange of lipids between LDs (63), leading to a decrease in the number of LDs and an increase in LDs volume (57, 58, 61). Currently, FSP-27 has been shown to have anti-lipolytic activity (56–58, 61, 64–66). In cancer cells, FSP-27 is also altered due to changes in lipid metabolism and is prognostic for lung and pancreatic adenocarcinoma (10, 67–70). Furthermore, Vishwajeet Puri and his team found that FSP-27 both directly inhibits ATGL ground activity and with synergistic EGR-1 inhibits *Pnpla2* expression (55, 56). The study found that FSP-27 binds directly to ATGL through its core structural domain, aa120-220, inhibiting its triglyceride degrading function and promoting triglyceride storage. On the other hand, FSP-27 enhanced the binding of EGR-1 in the -46/-34 region upstream of *Pnpla2* and, by doing so, inhibited *Pnpla2* expression. Although the above two studies did not use tumor cell lines, combined with the existing results of altered FSP-27 expression in tumor cells, it is reasonable to believe that the FSP-27/EGR-1/ATGL axis is also present in some cancer cells (**Figure 4**). It also needs to be further investigated whether hypoxia induces altered FSP-27 expression, to what extent it is involved in the regulation of FSP-27 expression, what role HIF-1 and HIG-2 play in the altered FSP-27 expression, and whether FSP-27 expression in tumor cells correlates with the availability of tumor cells to lipids.

## ATGL and Tumor Immune Microenvironment

Inflammation is a complex protective biological response to harmful stimuli (including damaged cells, pathogens, and irritants, etc.), involving immune cells, blood vessels, and molecular mediators. Inflammation can be broadly divided into acute and chronic inflammation. Macrophages, lymphocytes, and plasma cells play a major role in chronic inflammation, while neutrophils play a significant role in acute inflammation. In 1863, Rudolf Virchow proposed the hypothesis that cancer originates at the site of chronic inflammation (71–73). Currently, 15-25% of human cancers are due to chronic inflammation (73, 74). The cause of cancer from chronic inflammation is currently believed to be mainly the continuous production of ROS and reactive nitrogen species (RNS) by immune cells at the site of inflammation, which allows the accumulation of DNA damage and epigenetic alterations in normal cells, which eventually transform into cancer cells (71, 73, 75). In addition, inflammatory mediators produced in areas of chronic inflammation, prostaglandins, interleukin 1 beta, TNF-α, interleukin-8, interleukin-15, and chemokine (C-X-C motif) ligand 1 (CXCL1) also promote the proliferation and metastasis of tumor cells (71, 73, 75).

Tumor-associated macrophages (TAMs) are a vital link between chronic inflammation and cancer. Macrophages generally originate from bone marrow-derived blood monocytes (monocyte-derived macrophages) and yolk sac progenitor cells (tissue-resident macrophages), but the origin of human TAMs remains debatable (76). Like typical macrophages, TAMs are divided into M1 and M2 (77). M1 is thought to have pro-inflammatory and cytotoxic effects (antitumor), whereas M2 is thought to have anti-inflammatory (pro-tumor) and promote wound healing and tissue repair. In the tumor immune microenvironment, TAMs are more often considered to be M2 (78). TAMs can exert pro-tumor effects through the secretion of vascular endothelial growth factor, nitric oxide synthase, and nuclear factor κB (NF-κB) (79, 80), and the number of TAMs is negatively correlated with the prognosis of cancer patients (81). Currently, there is growing evidence that lipids have a significant impact on macrophage function and differentiation. The accumulation of triglycerides and LDs can be observed in inflammation-activated macrophages (82–86). Macrophage activation by Lipopolysaccharides (LPS) was found to inhibit ATGL-mediated triglyceride hydrolysis by upregulating HIG-2 in activated macrophages, ultimately leading to the accumulation of LDs (85). In contrast, upregulation of HIG-2 expression (31) and accumulation of LDs (87) was also found in TAMs. The accumulation of lipids in macrophages with altered lipid metabolism further affects their function and differentiation. Functionally, elevated FAs content promotes inflammatory responses in macrophages (88), while high LDs content attenuates them (83). In HIG-2-deficient bone marrow cells show a decrease in triglyceride content with an enhanced inflammatory response (85). The pro-inflammatory effect of FAs on inflammation may presumably be due to the fact that elevated intracellular FAs content increases the availability of FAs as precursors of inflammatory mediators, such as prostaglandins. However, a decrease in LDs content due to inhibition of triglyceride synthesis and an accompanying attenuated inflammatory response has also been observed in DGAT-deficient macrophages (89). This contradictory result may be due to the complexity of the inflammatory response in macrophages, which is also regulated by a variety of other factors, of which FAs are only one. In contrast, epidermal fatty acid binding proteins were found to upregulate lipid production of high levels of type-I interferons β (IFN-β) in TAMs, enhancing the tumor suppressive effect of TAMs (90). Interestingly, the effect of FAs metabolism on TAMs seems to depend on the FAs species (90), with increased metabolism of saturated FAs having pro-tumorigenic activity while increased metabolism of unsaturated FAs has tumor-suppressive activity (91). This may be due to the fact that in cells, unsaturated FAs produce more lipid peroxides relative to saturated fatty acids, which induce apoptosis. In terms of the differentiation of TAMs, lipid accumulation can promote the polarization of TAMs in the tumor microenvironment to the M2 (92). Furthermore, for example such as, prostate cancer-derived cathelicidin-related antimicrobial peptide (93), hypoxic cancer cell-derived oncostatin M and eotaxin (94), and myeloma derived MIF (95) can also independently polarize TAMs to the M2. In line with this, in ovarian cancer cells, ATGL and extracellular signal-regulated kinase 1/2 (ERK1/2) are involved in Pigment epithelium-derived factor (PEDF)-promoted polarization of macrophages to M1 (96). This suggests that lipids can also regulate the differentiation process of TAMs. In conclusion, the lipolytic process of triglycerides in TAMs plays a crucial role in both functional and differentiation processes of TAMs and ATGL, as the enzyme that hydrolyzes triglycerides must have a non-negligible influence in it. However, further elucidation of the specific mechanism of ATGL in TAMs is still needed.

## Different Roles of ATGL in Tumors

For different types of tumor cells, ATGL has different roles and can interact with various intracellular factors (**Table 1**). Therefore, we have summarized the roles of ATGL in different tumor cells in this section.

**Table 1:** Role of ATGL in different tumors and the interacting molecules.

Cancer type	Model	Outcome	Interacting molecular	References
Breast Cancer	C57BL/6J mice with *in situ* breast cancer	The deficiency of ATGL in lung neutrophils promotes breast cancer metastasis	prostaglandin E2	(97)
	4T-1, MCF-7, E0771, MDA-4175, AT-3			
	Murine 3 T3-L1 preadipocytes, MCF-10A	ATGL promotes tumor progression in breast cancer cells co-cultured with adipocytes	FABP5, PPARβ/δ, MAPK	(98)
	MDA-MB-231, T-47D	ATGL inhibition favors tumor cells	hGX sPLA	(99)
	BALB/cJ mice injected TS/A cells through the catheter	ATGL promotes tumor invasion	AMPK/acetyl-CoA carboxylase	(100)
	ZR-75-1, HMT-3522-T4-2, MCF-7, T47D, MDAMB-231, TS/A			
	MCF-7, MDA-MB231	ATGL knockdown attenuates the promotion of breast cancer cell proliferation and metastasis by adipocytes	CPT1A	(27)
	Patients and Clinicopathological Data	High peri-tumoral ATGL expression in obese patients	none	(101)
Lung Cancer	Frozen tissues of patients with adenocarcinoma/squamous cell carcinoma of the lung	ATGL produces pro-tumor effects	CHKα2	(102)
	HEK-293T, H322, H358			
	*Atgl*+/+ ctg, *Atgl*+/- ctg and *Atgl*-/- ctg mice	ATGL deletion induces tumorigenesis	none	(18)
	A549, HOP62, HOP92	ATGL knockdown inhibits tumorigenesis and metastasis and promotes apoptosis	G0S2	(103)
	A549	Depletion of ATGL facilitates cancer cell invasion	SRC	(104)
	LLC	ATGL inhibits tumor proliferation	AMPK-mTOR	(19)
Ovarian Cancer	IOSE80, ES2, A2780, HO8910, SKOV3	Upregulation of ATGL expression inhibits cancer cell growth migration and invasion	NEAT1, let-7g, MEST	(105)
	Macrophage	ATGL has a tumor-promoting effect	PEDF	(96)
Colorectal Cancer	Human colon tumor tissue	ATGL promotes tumorigenesis	ATG2B,PCK2,PGAM1,SPTLC2,IGFBP1,ABCC3,MYC,MUC2	(106)
	Colon and colonic tumors of high-fat-diet obese mice			
	HT29, HCT116, W620			
	C26, CT26	ATGL inhibits tumor proliferation	AMPK-mTOR	(19)
	HCT116, DLD-1	ATGL inhibits tumor proliferation	HIG-2	(34)
	HT29	Overexpression of ATG in prostate cancer cells	G0S2	(107)
Prostate Cancer	BPH1, CAFTD1	ATGL possess tumor suppressive effect	EPHB2	(7)
Melanoma	B16-F10	ATGL inhibits tumor proliferation	AMPK-mTOR	(19)
Liver Cancer	HepG2	ATGL inhibits tumor proliferation	AMPK-mTOR	(19)
	HepG2, Hep3B	ATGL promotes tumor proliferation	p-AKT	(21)
	Human HCC samples	ATGL inhibits tumor proliferation	PPAR-α/p300, p53	(108)
	C57BL/6 mice with Diethylnitrosamine			
	HepG2, Hep3B, Huh7.5			
	HCC tissues of patients	High expression of ATGL in HCC promotes tumor proliferation	lnc RNA NEAT	(109)
	HepG2, Huh7, SKHep-1, HCCLM3			
Prostate Cancer	CAFs, LNCaP, PC3	Low expression of ATGL in CAFs	PEDF, MTOC	(110)
	LNCaP	The knockdown of ATGL impeded the proliferation and invasion	ABHD5	(111)
	LNCaP	Overexpression of ATGL in prostate cancer cells	ABHD5	(107)
Cervical Cancer	HeLa	ATGL inhibits tumor proliferation	HIG-2	(34)
Renal Cancer	Caki-1, ACHN	ATGL inhibits tumor proliferation	HIG-2	(34)
Liposarcoma	ATGL KO mice	Knockdown of ATGL promotes cancer formation	HSL, GPNMB, G0S2	(47)
Pancreatic Cancer	Patients and clinicopathological data	Increased ATGL expression is associated with increased adiposity and stromal proliferation in patients with PDAC	none	(8)

### Breast Cancer

Based on the previous description, the expression of ATGL in tumor cells in a hypoxic microenvironment is usually decreased to reduce the intracellular FFAs. In contrast, low intracellular FFAs in human cells are generally associated with reduced FAO. Therefore cells are likely to respond to the hypoxic environment in this way. FAO and ATGL have often been found to have a promotional effect on breast cancer. The pro-oncogenic effect of FAO suggests that breast cancer cells require more FAs (112). In addition, the activation of FAO is often associated with an increase in the metastatic capacity of breast cancer cells (100). Breast cancer cells can induce the release of large amounts of FAs from nearby adipocytes, which they then store in the form of triglyceride, and later promote the occurrence of non-coupled FAO through the high expression of ATGL, ultimately increasing their metastatic ability (100). It seems that a large amount of lipid is prepared for the upcoming cancer cells at the site of breast cancer spread. In a mouse model of breast cancer lung metastasis, ATGL expression is suppressed in neutrophils located in the lungs of mice, leading to lipid accumulation in neutrophils (97). Lipids stored in neutrophils are transferred to breast cancer cells that have spread there *via* the giant cellular drinking-lysosome pathway, promoting their proliferation and survival (97). Mechanistically, Src is involved in FAO-induced metastasis in breast cancer cells (113). In addition, mitochondrial carnitine palmitoyltransferase 1a (CPT1A) was also aberrantly expressed in breast cancer cells (114, 115). ATGL is not the only lipase upregulated in breast cancer cells, where intracellular Lipase member H (LIPH) was also highly expressed in cancer cells from breast cancer patients (116). LIPH has also been found to have the ability to increase the metastatic ability of breast cancer cells (117). However, one study found that when G0S2 was knocked down in MDA-MB-231 cells, the proliferation, metastasis, and invasive ability of cancer cells were reduced (118). In triple-negative breast cancer (TNBC), sPLA can induce polyunsaturated fatty acids (PUFAs) to generate triglycerides or LDs, thus avoiding oxidative stress caused by PUFAs and thus promoting cancer cell survival. sPLA can also inhibit the activity of ATGL and prevent the degradation of PUFAs from triglycerides (99).

### Lung Cancer

In lung cancer, the effect of ATGL in cancer cells is ambiguous. Triglyceride accumulation in LDs and higher levels of cellular phospholipids and bioactive lipid species (lysophospholipids and ether phospholipids) were found in A549 lung cancer cells with knockdown of ATGL (104). A549 cancer cells with concomitant knockdown of ATGL had a more remarkable ability to migrate, associated with elevated levels of phosphorylated Proto-oncogene *Src*. This mechanism was also observed in breast cancer cells (113). Although different from the elevation of ATGL in breast cancer cells, elevated lipid levels are a common feature of both. However, in a mouse model of lung cancer, high expression of ATGL can promote tumor development (103, 119). The reason for this inconsistent result at the cellular and animal level may be the difference in the microenvironment in which the tumor cells are located. Compared to 2D cultured cells, the metabolic profile of 3D cultured ATGL-KO A549 cells and their microenvironment more closely resembled the actual situation *in vivo* (9).

### Hepatic Carcinoma

ATGL in hepatocellular carcinoma cells (HCC) appears to be more involved in cancer cell proliferation than metastasis. Overexpression of ATGL in hepatocellular carcinoma cells promotes phosphorylation of p-Akt, which promotes cancer cell proliferation but does not affect the metastatic ability of cancer cells (21). Similarly, the long non-coding RNA *NEAT1* driven cancer cell proliferation lipolytically by inducing ATGL expression in a mouse model of *in situ* hepatocellular carcinoma (109). However, in another human-derived HCC sample and induced mouse liver cancer model, ATGL expression was lower in the human HCC and mouse liver cancer models than in control tissues. The proliferation rate of HCC was negatively correlated with ATGL expression. This phenomenon was attributed to the upregulation of the tumor suppressor p53 due to ATGL upregulation, mediated by the PPAR-α/p300 axis (108).

### Pancreatic Cancer

In pancreatic cancer, tumor progression is closely related to lipid content and accessibility. Immunohistochemical analysis of 44 tissue samples from patients with PDAC revealed that ATGL expression was elevated in the tumor stroma of obese patients with PDAC. Still, ATGL content did not significantly correlate with tumor size and histological grade, so increased ATGL might be a critical factor in obesity-induced PDAC (8). In addition, increased adipocyte infiltration in the pancreas and hypertrophy of peritumor adipocytes, and increased levels of tumor tissue LDs formation-related proteins Mannose-6-phosphate receptor binding protein 1 (TIP-47) and Adipose differentiation-related protein (ADRP) were also found in EL-KrasG12D/PEDF deficient mice (120). EL-KrasG12D/PEDF deficient mice develop a more aggressive phenotype of PDAC. Notably, ATGL levels decrease with PEDF knockdown, suggesting that ATGL in pancreatic cancer may not have the same pro-metastatic ability as in breast cancer. Similarly, in another PDAC mouse model, inhibition of ATGL did not alter the difference in triglyceride abundance between *Hilpda* wild-type and knockdown cells (32). Therefore, in PDAC, ATGL may not be a critical factor in tumor proliferation and metastasis but might be a key factor in tumor formation.

### Colorectal Cancer

Like its role in breast cancer, ATGL can produce tumor-promoting effects in colorectal cancer. In colon cancer cells and colon cancer stem cells, obesity can promote ATGL-mediated LDs utilization for tumor development (106). Mechanistically, it was found that the promotional effect of ATGL on colorectal cancer was achieved by degrading triglycerides and encouraging the expression of genes related to sphingolipid metabolism and CoA biosynthesis (20). Although ATGL is associated with sphingolipid metabolism, ATGL does not regulate the expression of proteins related to sphingolipid metabolism.

### Prostate Cancer

ATGL has no beneficial effect on prostate cancer. DGAT1, ABHD5, and ATGL are overexpressed in prostate cancer cells compared to peripheral blood mononuclear cells, and inhibition of DGAT1 and ABHD5 was found to lead to prostate cancer cell death (107). In contrast, ABHD5 has also been found to have an inhibitory effect on the proliferation and invasion of prostate cancer cells (111), and they also noted that ABDH5 regulates prostate cancer cells independently of ATGL (121). The role of ATGL in prostate cancer cells is also regulated by ephrin B2 receptor (EPHB2). EPHB2 acts as EPHB2, a suppressor of prostate cancer cells, is able to exert its tumor suppressive effect by inhibiting the activity of lipogenic factors DGAT1, DGAT2 and promoting the lipolytic factor ATGL, PEDF (7). The development of prostate cancer cells may also be related to the surrounding environment. It has been found that the expression of ATGL and PEDF is lower in prostate cancer-associated fibroblasts compared to primary human normal prostate fibroblasts (110). It suggests that there may be more lipids stored in prostate cancer-associated fibroblasts to facilitate the development of prostate cancer cells.

## Perspective

In this review, we have endeavored to summarize the recent research progress on regulating ATGL-mediated lipid metabolism pathways in tumor cells and the implications of ATGL in different cancer types. The expression and activity of ATGL, a key enzyme of lipid metabolism in cells, directly affect whether the intracellular energy source is glucose or fatty acids. Because of the hypoxic conditions in tumors, most tumor cells tend to attenuate fatty acid oxidation, which reduces oxygen consumption and adapts itself to the hypoxic microenvironment. Hypoxia induces the expression of HIF-α and HIG-2, which in turn promotes the expression of PPAR-γ, the intracellular protein responsible for lipid anabolism. G0S2, as a PPAR-γ target gene, directly inhibits the activity of ATGL. In addition, HIG-2 was also found to inhibit ATGL activity and directly reduce the intracellular lipolytic process. Although this process has been validated only in adipocytes, there is reason to believe that this approach plays an equally important role in adapting tumor cells to the hypoxic microenvironment. Based on these two pathways, ATGL is expected to express low in tumors and negatively affects tumor development. In practice, however, it was found that ATGL is not lowly expressed in all cancer cells as not all cancer cells have a hypoxia-induced inhibition of fatty acid oxidation. In particular, ATGL levels were much higher in breast cancer cells than in normal cells. The active intracellular FAO also increased the metastatic ability of breast cancer cells. This suggests that cancer cells’ rapid proliferation and metastasis possibly need FAO, not glycolysis, to provide sufficient energy. Studies also revealed that the pro-tumorigenic effect of ATGL is often correlated with whether the location of tumorigenesis has access to large amounts of lipids. For example, breast cancer is surrounded by cells containing large amounts of lipid adipocytes (97), and pancreatic cancer stroma contains large quantities of lipids (8). However, it is essential to note that endogenous and exogenous fatty acids may bring about different or even opposite effects on cancer cells, according to the analysis of Marteinn Thor Snaebjornsson et al. (122). Therefore, more studies are needed to explore the impact of exogenous lipids on the role of ATGL in tumors.

Alterations in ATGL are the consequence of cellular carcinogenesis but not the inducement like proto-oncogene *Ras*. Tumor cells adjusting ATGL are more like a strategy to adapt to hypoxia. Nowadays, there are many studies on the role of ATGL in cancers, but there is a lack of studies on transcription factors that target *Pnpla2* on the changes in the ubiquitination process of ATGL in tumor cells. Therefore, more studies are needed to investigate the alteration of ATGL expression and degradation in tumor cells compared to normal cells, contributing to the development of antitumor drugs targeting related proteins in the future.

## Author Contributions

RZ and JM wrote the manuscript. SY, WL, LS, JZ, and JC made the figure and tables. DX contributed to the conception of the review. NL and BL check the corrected manuscript. All authors listed have made a substantial, direct, and intellectual contribution to the work and approved it for publication.

## Conflict of Interest

The authors declare that the research was conducted in the absence of any commercial or financial relationships that could be construed as a potential conflict of interest.

## Publisher’s Note

All claims expressed in this article are solely those of the authors and do not necessarily represent those of their affiliated organizations, or those of the publisher, the editors and the reviewers. Any product that may be evaluated in this article, or claim that may be made by its manufacturer, is not guaranteed or endorsed by the publisher.
